# Printable-Microencapsulated Ascorbic Acid for Personalized Topical Delivery

**DOI:** 10.1021/acsabm.3c00648

**Published:** 2023-11-20

**Authors:** Lapporn Vayachuta, Meyphong Leang, Jareerat Ruamcharoen, Raweewan Thiramanas, Sagaw Prateepchinda, Panida Prompinit, Sakkarin Du-a-man, Sutthinee Wisutthathum, Neti Waranuch

**Affiliations:** † National Nanotechnology Center (NANOTEC), National Science and Technology Development Agency (NSTDA), Khlong Luang, Pathum Thani 12120, Thailand; ‡ Faculty of Science and Technology, Prince of Songkla University, Muang, Pattani 94000, Thailand; § Cosmetics and Natural Products Research Center, Faculty of Pharmaceutical Sciences, Naresuan University, Phitsanulok 65000, Thailand

**Keywords:** personalized topical delivery, printable microencapsulation, ascorbic acid, transdermal delivery, laser printer

## Abstract

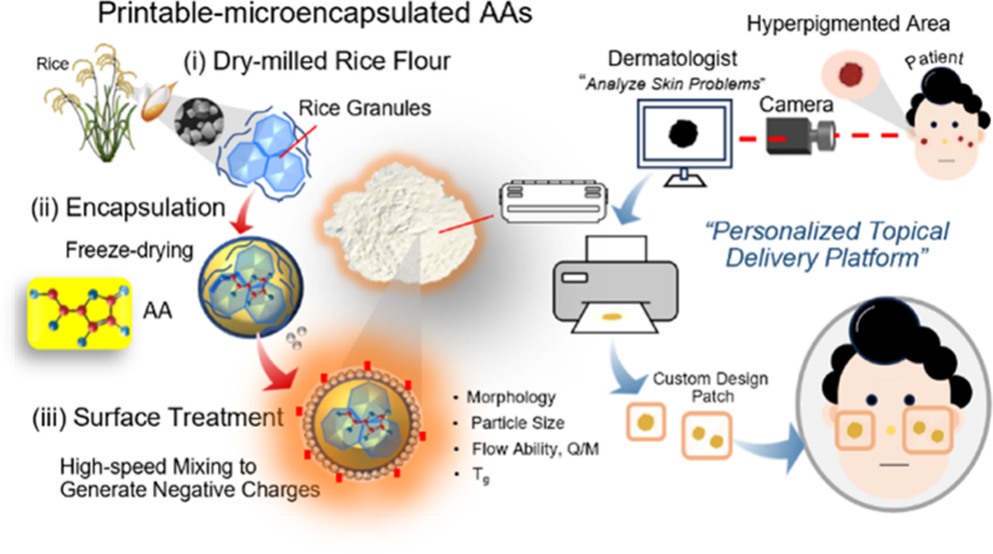

This study presents the successful development of printable-microencapsulated ascorbic acid (AA) for personalized topical delivery using laser printing technology. Rice flour with a 10% AA content was selected as an encapsulation material. Hydrophobic nanosilica was used to create negative electrostatic charges on the microencapsulated surfaces via a high-speed mixture. This process facilitated the microencapsulated AA fabrication using a commercial laser printer and produced a well-patterned design with some minor print defects, such as banding and scattering. The amount of encapsulated AA per area was 0.28 mg/cm^2^, and the RGB color code was 0,0,0. An emulsion carrier system comprising pentylene glycol (P5G) or diethylene glycol monoethyl ether (DEGEE), Tween 20, oleic acid, and deionized (DI) water at a ratio of 20:30:30:20 was developed to enhance AA transmission into the skin. The Franz diffusion cell technique was used to investigate topical absorption on Strat-M membranes using P5G and DEGEE as enhancers. The steady-state fluxes were 8.40 (±0.64) and 10.04 (±0.58) μg/h/cm^2^ for P5G and DEGEE, respectively. Cytotoxicity tests conducted on fibroblast cells revealed low cytotoxicity for the encapsulation products and carriers.

## 1 Introduction

The development of personalized drug delivery via two-dimensional (2D) and three-dimensional (3D) printing techniques has shown great potential for optimizing patient therapy by tailoring drug dose, dosage, and release based on individual needs.^1−6^ Among the various 3D-printing methods, extrusion-based technologies, such as fused filament fabrication and semisolid extrusion (SSE), have been extensively investigated owing to their versatility, precision, feasibility, and cost-effectiveness. These methods are widely applied in many areas, such as personalized drug dosing, including flexible dose tablets for immediate and extended release^7^ and polypills;^8,9^ personalized topical treatment devices such as nose-shaped masks;^10^ medical devices such as vaginal rings;^11,12^ drug-eluting scaffolds;^13^ biodegradable patches for pancreatic cancer;^14^ and patches for transdermal drug delivery,^15,16^ such as microneedles.^17,18^ Notably, 3D-printing techniques might improve the precision dosing and safety. Nowadays, these platforms are being advanced by connecting them to digital devices, such as 3D scanners and smartphones. A prospective personalized nose-shaped mask was designed by Goyanes et al. The process includes scanning the treatment location of the patient and using 3D printing to print out a mask that fits the device to the nose of the patient.^10^

For 2D-printing techniques, an innovative approach using inkjet-printed quick response (QR)-encoded dosage forms containing a personalized dose of the drug as well as information relevant to the end-user in a QR pattern, readable by a standard smartphone, was recently published.^19,20^ Öblom et al. developed a potential technique to produce personalized medicine via inkjet printing, enabling the precise deposition of a desired volume of drug-loaded ink onto edible substrates.^20^ The flexible nature of the technique facilitates the easy manipulation of the printed pattern, enabling the manufacturing of drug products with unique information-rich patterns, e.g., QR codes.

In 2019, P&G introduced the Opté Precision Skincare System,^21^ a compact beauty tool designed to rapidly eliminate imperfections on facial skin, including blemishes, freckles, and scars. The device analyzes skin pigmentation and employs 120 inkjet nozzles to precisely inject the correct amount of foundation or tinted moisturizer onto the affected areas, resulting in a flawless and natural-looking complexion. Moreover, the “Mink” 3D makeup printer^22^ has recently been introduced. This device has the remarkable ability to instantly convert any image to wearable makeup. The printer can produce an astonishing 16.7 million makeup colors in just 15 s per sheet, making it a highly efficient and convenient option for makeup enthusiasts.

In the future, the application of 2D- and 3D-printing technologies is expected to increase in popularity and significance in beauty devices and personalized drug delivery.^23^ These technologies have the potential to create customized solutions that address the specific needs and preferences of each individual. By leveraging the precision and flexibility of printing techniques, it is possible to produce beauty devices and drug delivery systems that are tailored to a particular user, resulting in more effective and efficient treatments. Using printing technologies offers new avenues for developing safe and user-friendly personalized solutions.

However, personalized drug delivery fabricated via existing technologies faces several limitations, such as degradation of AI due to heat or moisture during the fabrication process and nozzle clogging. Two dimensional–based laser printing technology may be used to address these issues. An electrophotographic process generally involves five steps: imaging, inking, toner transfer, fusing or fixing, and cleaning. Imaging is achieved by charging a photoconductive surface, usually a drum or a belt, with a uniform electrostatic charge from a corona or charge roller. The photoconductive surface charge is positive for most inorganic photoconductors and negative for organic photoconductors (OPCs). Then, the charged drum is exposed to light to produce a latent electrostatic image on the photoconductor, partially discharging the uniform charge on the surface. Inking occurs when the toner particles are transferred from the developer to the photoconductor via electric potential differences. The toner particles selectively adhere to the discharged area of the photoconductor but repel the charged area. After inking, a latent image became visible owing to the applied toner. Most toners are made of styrene copolymers, such as styrene coacylate and styrene–butadiene–acrylic acid^24^ as binders, pigments, charge transfer agents (CCAs), wax, silica, and other additives.^25^ Normally, the toner can be prepared via polymerization or pulverization. In pulverization, all chemicals are fused and kneaded in a preadjusted binder resin and then milled and classified. Properties of toners should include a low softening point (*T*_g_ ∼ 63 °C) and sharp melting characteristics to save energy during the fixing process on the paper substrate. The flowability of the toner was controlled using charge control agents (CCAs) and additives.^26^

In the present work, we first introduce a novel platform to accurately fix microencapsulated ascorbic acid (AA) to desirable locations. This comprises modifying the surfaces of the microencapsulated particles and transferring them onto a specific area using a commercial laser printer following a map generated by using an image processing system. Ascorbic acid was selected as a model of water-soluble AI. Its activity is easily detected by conventional methods, such as DPPH assays, and it is widely used to treat hyperpigmented areas on the skin.^27^ Polysaccharides from rice flour were also selected as encapsulating materials because their granule morphology and size resembled that of a commercial toner. Specifically, the water-soluble portion of rice flour was used as an encapsulating material because it could swell or blend with AA in an aqueous solution. Moreover, it could be recoated on the rice granule or form a new particle during mixing in the encapsulation process. A significant advantage of this approach is that the original morphology of a rice granule can be used as the core of the AA shell. Furthermore, the soluble components of the flour adhere to the AA surfaces, eliminating the need for the addition of another polymer. In addition, this method does not require a subsequent drying process using hot gases, such as spray dryers,^28^ to convert the liquid or slurry polymer into micropowders. Therefore, the thermal decomposition of AA during the encapsulation process was minimized.

**Figure 1:**
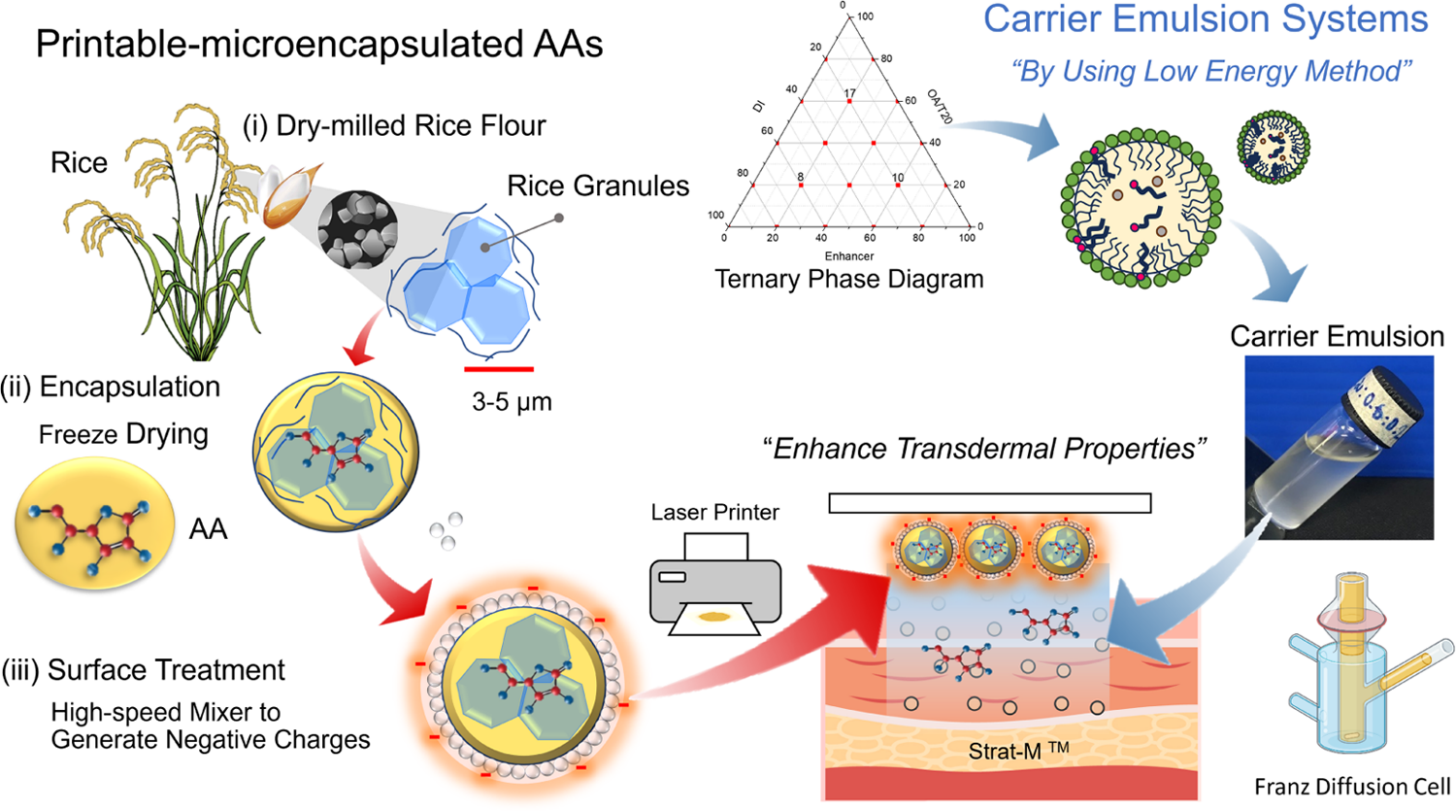
Schematic view for fabrication of printable-microencapsulated ascorbic acids and carrier emulsion systems.

The schematic diagram of this work is shown in Figure 1. The fabrication of printable-microencapsulated AAs consists of three steps: (i) dry-milling the rice gain to obtain fine particles, (ii) encapsulating AAs, and (iii) treating their surface with hydrophobic silica to create negative charges (−*q*) on the encapsulated particle surfaces without using any charge transfer agent. Fumed silica has been widely used to treat the surfaces of powders to improve their flowability.^29^ To prepare printable encapsulated particles, three criteria must be considered: (i) the particle size should be approximately 5–7 μm with unimodal distribution, (ii) the shape of the particle should be a sphere- or potato-shaped^30^ to minimize contact with the organic photoconductor (OPC) drum, and (iii) the encapsulated particles should have suitable electrostatic charges on their surface, depending on the type of laser printer. In this study, a commercial laser printer, a Brother HL-2130, was used to fabricate the encapsulated AAs. This type of printer requires a negatively charged toner. First, the specification of a commercial toner was evaluated. It provided round-shaped particles with an average size of 6.7 μm and a negative charge of −44 μC/g. The morphology, particle size, particle size distribution, flowability, bulk density, and encapsulation efficiency of the microencapsulated AAs were investigated. A commercial laser printer was used in the fabrication step by filling prepared microencapsulated AAs into a clean cartridge. After designing the target pattern with an image processing program, the transfer ability, patterning ability, and defects on the different flexible substrates, such as PET sheets, were observed. Finally, to increase the released amount and penetration level of the AAs from the microencapsulated particles into the target area, carrier solution systems were designed and applied. Our delivery systems offer advantages, such as the ability to control the amount and release rate of active compounds and precisely transfer them onto a designed target, reducing the risk of active overdose.

## 2 Materials and Methods

### 2.1 Materials

Rice flour was obtained from Novolife Co., Ltd. It was analyzed according to the Association of Official Analytical Chemists (AOAC, 1990) methods and found to contain 6.84 ± 0.03% protein, 0.62 ± 0.03% fat, and 0.45 ± 0.03% ash content. White rice grains were milled using a jet mill to obtain uniform and fine particles. The rice flour was kept in a plastic bag at room temperature until it was used. l-Ascorbic acid (reagent grade) was purchased from Sigma-Aldrich. 1,1-Diphenyl-2-picrylhydrazyl radical (DPPH, 97.0%) was purchased from TCI. Ethanol (95%) was purchased from Fisher Chemicals. Glycerin (99.5%) was purchased from Thai Glycerine Co., Ltd., and pentylene glycol (100%) was purchased from Activon Co., Ltd. Diethylene glycol monoethyl ether (for analysis) was purchased from Carlo Erba. Oleic acid (extra pure) was purchased from QRëC. Poly(ethylene glycol) sorbitan monolaurate (Tween 20) was purchased from Chemipan Co., Ltd. All chemicals and reagents were used as received without further purification.

Fluorescein isothiocyanate isomer I (Fit C) with an excitation maximum of approximately 491 nm and an emission maximum at 516 nm (green) and Nile Red with an excitation maximum of approximately 515 nm (green) and an emission maximum of approximately 585 nm (yellow–orange) were purchased for microscopy from Sigma-Aldrich. A Strat-M membrane was purchased from Merck Millipore. Phosphate-buffered saline (PBS, pH 7.4) powder was purchased from Sigma-Aldrich. Hydrophobic-fumed silica AEROSIL R202 and AEROSIL R812S (primary particle size of ∼7–40 nm) were purchased from Evonik, Korea. HDK-H15 was purchased from Wacker Chemie AG. The specifications of hydrophobic silica are shown in Table S1. A poly(ethylene terephthalate) (PET) film with a thickness of ∼120 μm was used as a print substrate. The patterns were fabricated using a commercial laser printer (Brother HL-2130), and 10 g of the encapsulated powders was placed into the clean cartridge without further modification.

### 2.2 Characterization

The amount of AA was determined via an antioxidant capacity study using a DPPH assay in a 96-well plate. Before the measurement, the sample plate was shaken for 3 s, and the ultraviolet (UV) absorbance was recorded at 517 nm wavelength in triplicate for each test concentration using a microplate spectrophotometer (BioTek, Power wave XS2).

Fourier-transform infrared (FTIR) spectra were obtained using Fourier transform infrared spectroscopy (Nicolet IS50, attenuated total reflection (ATR) mode) over a scanning range of 500 to 4000 cm^–1^ with 64 scans. Powder X-ray diffraction patterns were recorded with a Bruker D8 advance diffractometer equipped with Cu Kα radiation (λ = 0.15406 nm) operated at 40 mA and 40 kV. The scan rate was 2θ = 0.02°/S over a range of 3 to 70°. The crystallinity degree (CI) of the sample was determined from X-ray diffraction (XRD) spectra.^31^

The surface morphology of the particles was examined using scanning electron microscopy (SEM; Hitachi: S3400N). To study the cross-sectional morphology, the particles were mixed with commercial cyanoacrylate glue and placed in a silicone mold. The sample was left to polymerize in the presence of moisture from the air at 25 °C/60% relative humidity (RH) for 24 h. Then, residual monomers were removed by vacuum. The samples were cut and coated with a thin layer of gold using a sputtering technique.^32^ The printing quality of the encapsulated powders on the PET substrate was examined by using an optical microscope (Olympus BX Series).

Micro energy-dispersive X-ray (EDX) fluorescence (XRF: Orbis PC) was used to verify the composition of the encapsulated powders as well as their contamination. Here, 1 g of the sample was placed over a thin film lining a 10 mL polypropylene cup and then mounted inside an EDX-7000 spectrometer. The instrument was equipped with an X-ray tube using a Rhodium (Rh) target and operated at 2.5–3.5 kV via PCEDX-Navi software. The analysis was conducted 3 times. The elemental compositions of all samples were determined under a room temperature air–based atmosphere. The analytes were then assessed using a collimator with a diameter of 0.5 mm and a live acquisition time of 60 s.^33^

The average particle sizes of the samples were determined by using a laser particle analyzer (Masterizer: Horiba, LA-960) in dry mode with three replications. The charges of the encapsulated powders were examined by performing three electrical single-particle aerodynamic relaxation time (E-SPART) measurements and analyzed by Hosokawa Micro Co., Japan.^34^ The viscosity of the carriers was determined at 25 °C by using a microVISC viscometer (RheoSense Inc., San Ramon, CA) in three replicates. To identify the types of carrier emulsion, an oil phase was stained with a Nile Red dye in a concentration of 0.01 mg/mL, while the water phase was stained with a Fit C dye in a concentration of 0.02 mg/mL. Then, 100 μL of formulated emulsions was added into a 96-well plate, and the phase behavior was investigated using high content analysis (PerkinElmer, Model opera phenix). A hand-held conductivity meter (Seven2Go Cond meter S7, Mettler Toledo) was used to measure the conductivity of the prepared emulsions in three replications. Thermogravimetric analysis (TGA) was performed using an STA 449 F5 Jupiter (NETZSCH, Germany) device under a nitrogen atmosphere with a flow rate of 40 mL min^–1^. Each sample (∼1 mg) was placed in a crucible and then heated from 20 to 700 °C at a heating rate of 10 °C min^–1^. Differential scanning calorimetry (DSC) analysis was performed using a NETZSCH DSC 214 Polyma. Then, the samples (1 mg each) were packed in DSC aluminum pans with closed lids. A heating scan was conducted from 20 to 250 °C at a heating rate of 10 °C min^–1^.

### 2.3 Preparation of Printable Encapsulated AAs in Rice Flour

#### 2.3.1 Preparation of Encapsulated AAs in Rice Flour

An AA solution of 10% was freshly prepared and well mixed with 90 g of rice flour in a 500 mL round-bottom flask. To start the freeze-drying process for 48 h, the white paste sample was immersed in liquid nitrogen for 5 min until it completely froze. Then, 30 g of dried samples was crushed to obtain a fine powder using a high-speed blender, Electrolux, at 2700 rpm (speed Number 3) for 1 min. The powder samples were sieved to obtain the particle size ranges of ≤125 μm, and the samples were kept in plastic bags until usage.

#### 2.3.2 Charing of Encapsulated AAs in Rice Flour

28.5 g portion of encapsulated AAs in rice flour was mixed with 1.5 g of nanosilica using a high-speed blender, Electrolux, at 2700 rpm (speed Number 3) for 1 min. Then, the samples were kept in a plastic bottle before usage. The sample compositions obtained in this study are shown in Table 1.

**Table 1:** Sample Compositions

samples codes	rice flour (g)	AA (g)	silica (g)
R	100		
10C-R	90	10	
1R202-10C-R	90.0	8.99	1
3R202-10C-R	88.18	8.81	3
5R202-10C-R	86.37	8.63	5
5R812S-10C-R	86.37	8.63	5
5HDK-10C-R	86.37	8.63	5

### 2.4 Study Flow Properties

#### 2.4.1 Angle of Repose

The angle of repose corresponds to the angle formed by the horizontal base of the bench surface and the edge of a cone-like pile of sample powders. A plastic funnel, which has an orifice size of 8 mm and a height of 140 mm, was used in this study. The funnel was fixed at 3 cm above the bench surface. Then, approximately 5 g of sample was added into the funnel, and the height of the powder sample forming the cone (*h*) and the radius (*r*) of the base were measured in three replications. The angle of repose (θ) was calculated as given in eq 1
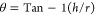
1

#### 2.4.2 Bulk and Tapped Densities

The bulk density (ρ_bulk_; g/cm^3^) is usually determined according to the method suggested by Shah et al.^35^ and includes measuring the volume of 30 g of powder in a 100 mL graduated cylinder. The volume of the powder (*V*_o_) was collected in triplicate as described by eq 2. The cylinder was adjusted 5 cm above the bench surface using a ring clamp, and the powder was allowed to free-fall. The tapped volume (*V*_f_) was measured after applying mechanical tapping 200 times. The final tapped volume was collected in three replicas. Then, the tapped density (ρ_tapped_, g/cm^3^) was evaluated according to eq 3
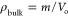
2
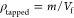
3where ρ_bulk_ represents the bulk density and ρ_tapped_ represents the tapped density.

#### 2.4.3 Carr’s Compressibility Index and Hausner Ratio

The bulk and tapped densities were used to calculate Carr’s compressibility index or Carr’s index (CI) eq 4(36) and the Hausner ratio eq 5. The flowability characteristics of the powder samples are shown in Table S2.
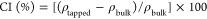
4
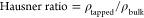
5

### 2.5 Releasing Behavior of AAs

In this study, a carrier solution was used to release AAs from the particles, which were then passed through an artificial membrane representing human skin (Strat-M membrane). Three types of enhancers of different log *P* values were selected to form an emulsion with Tween 20 and oleic acid. These enhancers included glycerin (G), pentylene glycol (P5G), and diethylene glycol monomethyl ether (DEGEE). The log *P*,^37^ hydrophile–lipophile balance (HLB), and viscosity of the materials are shown in Table S3.

#### 2.5.1 Preparation of the Carriers

Three formulas with different types of enhancers were prepared by using a low-energy method. The carrier compositions are shown in Table 2. All ingredients were well mixed using a multirotator (Multi Bio RS-24, Biosan) at room temperature for 1 h. The stability of the carriers was tested by performing centrifugation at 2260*g* for 10 min. The phase separation of the carriers after centrifugation was inspected by visual observation.

**Table 2:** Composition of the Carriers

carrier formulation	enhancer (mL)	T20 (mL)	OA (mL)	DI water (mL)	Total (mL)
#8	20	10	10	60	100
#10	60	10	10	20	100
#17	20	30	30	20	100

#### 2.5.2 Solubility of AA Powders in Carriers

The solubility of AA powders in various carriers was studied. To this end, 1 mL of carrier solution was added to 400 mg (or excess) of AA powders and mixed using a multirotator (Multi Bio RS-24, Biosan) at room temperature for 1 h in three replicas (*n* = 3). Undissolved AAs were separated using a centrifuge at 2260*g* for 15 min. Then, the supernatant was separated. The solubility of AA powders in carriers was studied by using a DPPH assay. To adjust the sample concentrations, the liquid was diluted until its concentration matched the AA standard curve. Then, 100 μL of the diluted sample was added to a 96-well plate and 100 μL of a 0.2 mM DPPH solution was added. The sample was kept in a dark place for 30 min. Then, absorbance at 517 nm was recorded,^38^ and the solubility of the AA powders in the carriers was evaluated using the standard calibration curve of AA. The %inhibition was calculated using eq 6
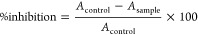
6

#### 2.5.3 AA In Vitro %Release Rate from Encapsulated Samples

The AA %release rate from the encapsulated samples was investigated. To that end, 1 mL of carrier solution was added to 100 mg of encapsulated samples and mixed using a multirotator (Multi Bio RS-24, Biosan) at room temperature for 1 h in three replicas (*n* = 3). The liquid part was separated using a centrifuge at 2260*g* for 15 min. Then, the supernatant was separated. To adjust the sample concentrations, the liquid was diluted until its concentration matched the AA standard curve. Then, 100 μL of the diluted sample was added to a 96-well plate, and 100 μL of a 0.2 mM DPPH solution was added. The sample was kept in the dark for 30 min, and the absorbance was recorded at 517 nm.

### 2.6 Transdermal Behaviors of AAs

Franz cells with a 1.767 cm^2^ orifice area were used in this study. A Strat-M membrane^39,40^ without presoaking was mounted on the flat flange of the Franz cells. Then, the donor compartment was attached by using a metallic clamp. After the donor compartment was attached, PBS buffer (pH = 7.4) was added as a receptor medium through the sampling port using a syringe until the cell was full. All cells had a receptor medium volume of 12 mL. Bubbles were removed by tilting and shaking the cell. The cells were stirred at 25 °C by using small magnetic stirrers. All cells were equilibrated for a minimum of 15 min before sample application. In each cell, 100 mg of encapsulated sample and 1 mL of carrier were placed in the donor chamber. Then, 300 μL of receiver fluid was withdrawn through the sampling port by using a syringe. Fresh PBS was added to maintain the volume of the receptor medium. The experiments were performed in two replicas (*n* = 2). 100 μL portion of the sample was added to a 96-well plate, followed by the addition of 100 μL of a 0.2 mM DPPH solution. The sample was kept in a dark place for 30 min, and the absorbance was recorded at 517 nm. The results were then plotted as accumulative concentration (μg/cm^2^) against time to construct a release graph, and the maximum flux (*J*_ss_) was determined from the slope of the plot.

#### 2.6.1 Permeation Rate

The corrected data are expressed as the cumulative AA permeation per unit of skin surface area^41^ as shown in eq 7
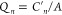
7

where *Q_n_* (μg) is the cumulative amount of AA per unit area. *C*′*_n_* is the corrected AA concentration in the *n*th sample, *C_n_* is the measured AA concentration in the nth sample, *C*′_*n*–1_ is the corrected drug concentration in the (*n*–1)th sample, *C*_*n*–1_ is the measured AA concentration in the (*n*–1)th sample, *V*_t_ is the total volume of receptor solution, *V*_s_ is the volume of the sample, and *C*′_1_ = *C*_1_.

*C*′*_n_* = *C_n_*(*V*_t_/*V*_t_ – *V*_s_)(*C*′_*n*–1_/*C*_*n*–1_).

*A* = 1.767 cm^2^.

#### 2.6.2 Steady-State Flux (*J*_ss_)

The cumulative amount of AA per unit area (*Q_n_*, μg) in a receiver chamber was plotted as a function of time (*t*, h), and the steady-state flux (*J*_ss_, μg/h/cm^2^) was calculated from the slope of the linear portion of the curve.

#### 2.6.3 Permeability Coefficient (*K*_p_)

The apparent permeability coefficient (*K*_p_, cm/h) was calculated according to eq 8.
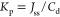
8where *C*_d_ is the AA concentration in the donor compartment.

#### 2.6.4 Diffusion Coefficient (*D*)

The diffusion coefficient (*D*, cm^2^/h) was calculated according to eq 9.
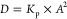
9where *K*_p_ is the permeability coefficient

*A* = 1.767 cm^2^.

### 2.7 Cytotoxicity Assay

Human fibroblast ATCC CRL2522 cells were resuspended in Dulbecco’s Modified Eagle’s Medium (DMEM, Gibco) containing 10% fetal bovine serum (Gibco), 1% l-glutamine (Gibco), and 1% penicillin/streptomycin (Gibco). The cells were cultured in a 96-well plate at 10,000 cells per well and incubated at 37 °C for 24 h. Then, the cells were treated with samples diluted in a DMEM medium and further incubated at 37 °C for 24 h. An untreated sample was used as a negative control, and 2.5% dimethyl sulfoxide (DMSO) was used as a positive control. The cell viability was determined by the CellTiter-Glo Luminescent Cell Viability Assay (Promega) according to the manufacturer’s protocol. After adding the reagent for 10 min, the luminescence signals corresponding to the amount of ATP molecules that represent the metabolically active cells were measured using a luminometer (SpectraMax L, Molecular Devices). The half-maximal inhibitory concentration (IC_50_) values, representing the concentration of particles that inhibit cell growth by 50%, were calculated by fitting a curve with nonlinear regression using a Quest Graph IC_50_ Calculator. Data measurements are presented as the mean ± standard deviation (*n* = 3). Student’s *t*-tests were performed, and the calculated *p* values were considered significant for *p < 0.05, **p < 0.01, and ***p < 0.001.

### 2.8 Biocompatibility Assay

Human fibroblast ATCC CRL2522 cells were cultured in a 96-well plate at 2000 cells per well and incubated at 37 °C for 24 h. Then, the samples diluted in DMEM complete medium were added to the cells and further incubated at 37 °C for 7 days without medium changing. Cell viability and membrane integrity were followed by the CellTiter-Glo Luminescent Cell Viability Assay (as described above) and Image-IT DEAD Green Viability Stain (Invitrogen) at 1, 4, and 7 days. Image-iT dye was used to determine plasma membrane permeability. The dye could not penetrate to the healthy cell membrane; however, it became permeant when the membrane integrity of cells was damaged. For membrane integrity assay, the culture medium was removed at the time of investigation. Then, the mixture of Image-IT dye diluted in DMEM complete medium was added. After that, the cells were incubated under normal cell culture conditions for 30 min and fixed with 4% paraformaldehyde in PBS at room temperature for 15 min. Finally, the fluorescence intensity was measured using a microplate reader (Synergy H1, BioTek) by an excitation at 488 nm and an emission at 515 nm. The membrane permeability of sample-treated cells was determined by comparing to the untreated cells. Normalized fluorescence intensity is reported as mean ± standard deviation (*n* = 4). Student’s *t*-tests were performed, and the calculated *p* values were considered significant for *p < 0.05, **p < 0.01, and ***p < 0.001.

### 2.9 In Vivo Skin Irritation Study

The acute cutaneous tolerance of the formulation and carriers on adult subjects was evaluated using a single patch test to determine the acute irritation potential of the formulation after a single application. The procedure of the investigating center, the protocol, the information sheet, informed consent, and the information concerning the investigational product (particularly its safety) were submitted to the Institutional Ethics Committee (Thailand). The study was approved on expedited approval with certificate no. EXP#1450. Subjects gave their informed and written consent. The inclusion criteria specified that subjects should be above 18 years old, preferably 20–60 years old, and could include males and females without previous intolerance or allergic reactions to this type of product. Additionally, subjects were required to have phototypes I to IV. Twenty-five microliter of samples were used as received and applied as occlusive patch types. The patch was applied on the subject’s scapular part of the back for 48 h. The mean cumulative irritation index (MCII) was graded by a dermatologist based on the occurrence of erythema and edema 30 min and 24 h after patch removal. A patch without the product was used as a negative control. The obtained index (maximum 6) allows us to arbitrarily classify the studied product according to the following scale: MCII < 0.25 = nonirritating (NI), 0.25 ≤ MCII < 0.50 = very slightly irritating (VSI), 0.5 ≤ MCII < 1 = slightly irritating (SI), 1 ≤ MCII < 2 = moderately irritating, and MCII ≥ 2 = irritating.

## 3 Results and Discussion

The composition of dry-milled rice was initially analyzed according to the Association of Official Analytical Chemists (AOAC, 1990) methods. It consisted of 6.84 ± 0.03 wt % protein, 0.62 ± 0.03 wt % fat, and 0.45 ± 0.03 wt % ash content. Carbohydrates were the major components in rice flour, including amylose and amylopectin. Their structure consists of long chains of glucose (Figure S1(a)). There are six levels of internal rice structures in each grain: the smallest level of individual branches, branched molecules, crystalline and amorphous lamellae, growth rings, granules, and the whole grain (Figure S1(b)).^42^ The microencapsulated particles in this study were prepared from finely ground rice grains, preserving their granular structure. After dissolving in water, filtering, and drying, the sample was separated into two parts of rice flour: an insoluble (R-ND) and a soluble (R-D) part. The percentage of the soluble part of rice flour was determined to be 9.7%, and it formed a yellow transparent film (Figure S2(a)). This part resulted from destruction of the crystalline structure. Leewatchararongjaroen et al. reported flour with lower crystallinity compared to that obtained with the wet-milling process.^43^ After drying, the R-ND agglomerated and was ground to a fine powder. The chemical and crystal structures of R, R-D, and R-ND were studied by using FTIR and XRD techniques.

The ATR-FTIR spectra of native rice flour (R) showed signals at 3300 cm^–1^ corresponding to OH stretching of two types of polysaccharides, amylose, and amylopectin. The peaks at 2890 and 2925 cm^–1^ were assigned to CH_3_, CH_2_, and CH stretching modes. The broad peak at 1643 cm^–1^ corresponded to a protein structure, and the peaks at 1000–1200 cm^–1^ were assigned to C–O, C–C, and C–O–H stretching and C–O–H bending modes (Figure S2(b)). Moreover, intramolecular hydrogen bonds in the starch structure were observed. A decreasing peak intensity at 1022 cm^–1^ was observed in R-ND, indicating a decrease of the amorphous part in their structure after dissolving in water (Figure S2(c)).

The structures of R and nonsoluble rice flour (R-ND) were further studied using XRD techniques. Single diffraction peaks at 15.4 and 23.3° and a doublet peak at 17.30° (Figure S2(d)) appeared. These peaks correspond to an “A type” crystalline structure with the following lattice parameters: *a* = 11.90 Å, *b* = 17.70 Å, *c* = 10.52 Å, and α = β = γ = 90°.^44^ The degrees of crystallinity (CI) of R and R-ND was calculated. It equaled 16.7 and 26.29%, respectively. The increase in CI in the R-ND structure confirmed that water could dissolve some amorphous parts of R.

### 3.1 Characterization of Printable Encapsulated Particles

A high-speed mixer was used to produce contact charging or tribocharging during mixing. The particles were charged by contacting each other. They exhibited positive or negative charges depending on the difference in the work function of the material.^45^ The blade of the mixer was made with a stainless steel 361. During mixing, contact between blade/silica particles, blade/encapsulated particles, and silica particles/encapsulated particles generates electron transfer on the surfaces. Higher work function (WF) materials enable electrons to lower the WF of the material to create negative charges (−*q*). The WF of stainless steel 361 is 4.92 to 5.06 eV, and the WF of silica is 5.00 eV. After mixing, the surface of the encapsulated polymer is loosely covered with silica particles, introducing physical forces such as an electrostatic interaction. These forces create a space between particles, improving flowability. The stability of the negative charges can be maintained on the surface because the polymer materials have high dielectric constants:^46,47^ 2.1 for AA, 3.0 for rice, 3.6–4.2 for silicon dioxide (SiO_2_), and 1 for dry air. Therefore, charge localization is limited. However, the charges on the surfaces can be reduced by moisture in the air. However, the reduction of the charges can be limited by high-carbon content silica. Thus, three types of silicas with different carbon contents, including AEROSIL R202, AEROSIL R812S, and HDK-H15, were used in this study. We observed that treating the surfaces of the silicas transformed the encapsulated powders into a fluid-like state (Figure S3).

**Figure 2:**
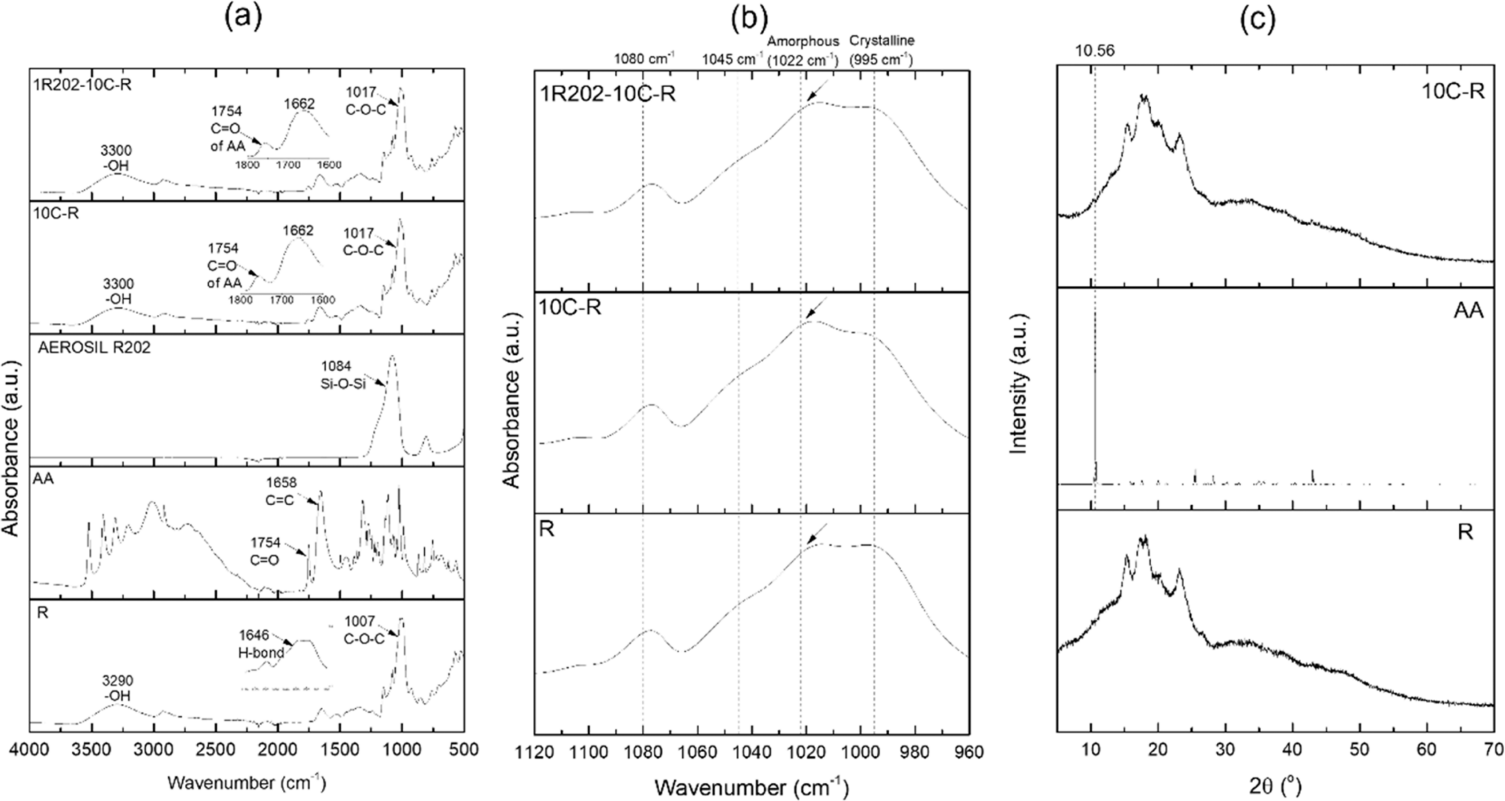
FTIR spectra of dry-milled rice flour (R), l-ascorbic acid powders (AA), hydrophobic silica (AEROSIL R202), 10% AA encapsulated in rice granules (10C-R), and 10C-R treated with 1% hydrophobic silica (1R202-10C-R): (a) 500–4000 cm^–1^, (b) 960 cm^–1^, and (c) XRD patterns of R, AA, and 10C-R.

FTIR spectra of encapsulated rice granules with and without hydrophobic silica-treated surfaces are shown in Figure 2. Both compounds exhibit similar peak positions. Therefore, only 10C-R and 1R202-10C-R were selected for this investigation. Figure 2(a) shows the FTIR spectra of 1R202-10C-R compared with those of the raw materials R, AA, and AEROSIL R202 silica. The spectra of 10C-R exhibited signals at 3300 cm^–1^, corresponding to the OH stretching of glucose units that are present in amylose and amylopectin in the starch granule. The peaks at 2890 and 2925 cm^–1^ correspond to the C–H stretching of CH_3_, CH_2_, and CH, whereas the peak at 1754 cm^–1^ corresponds to the C=O stretching of AA. The broad peak at 1662 cm^–1^ corresponds to the C=C stretching of AA and the intramolecular hydrogen bonds in the starch structure. Furthermore, the bands at 1084 cm^–1^ corresponding to the Si–O–Si stretching were not present in 1R202-10C-R owing to the low silica content on their surfaces (1%). A new peak, shifting from 1007 to 1017 cm^–1^ (Figure 2(b)), corresponding to the increasingly amorphous structure of the rice, was observed. The results confirmed that the main morphology of rice was not affected by this encapsulation method.

The structures of R, AA, and 10C-R were studied by using XRD (Figure 2(c)). The XRD patterns of AA exhibited diffraction peaks at 10.56, 17.54, 21.33, 25.41, 27.37, and 28.16°, corresponding to the crystalline structure of AA. After encapsulation, the XRD pattern of 10C-R containing 10% of AA exhibited peaks at 15.4 and 23.3° and a doublet at 17.30°. Major diffract peaks of most AAs at 10.5° could not be observed because the AA crystals were completely soluble and compatible with the soluble fraction of rice flour (9.7% of the total weight) to create the AA/starch amorphous phase.

**Figure 3:**
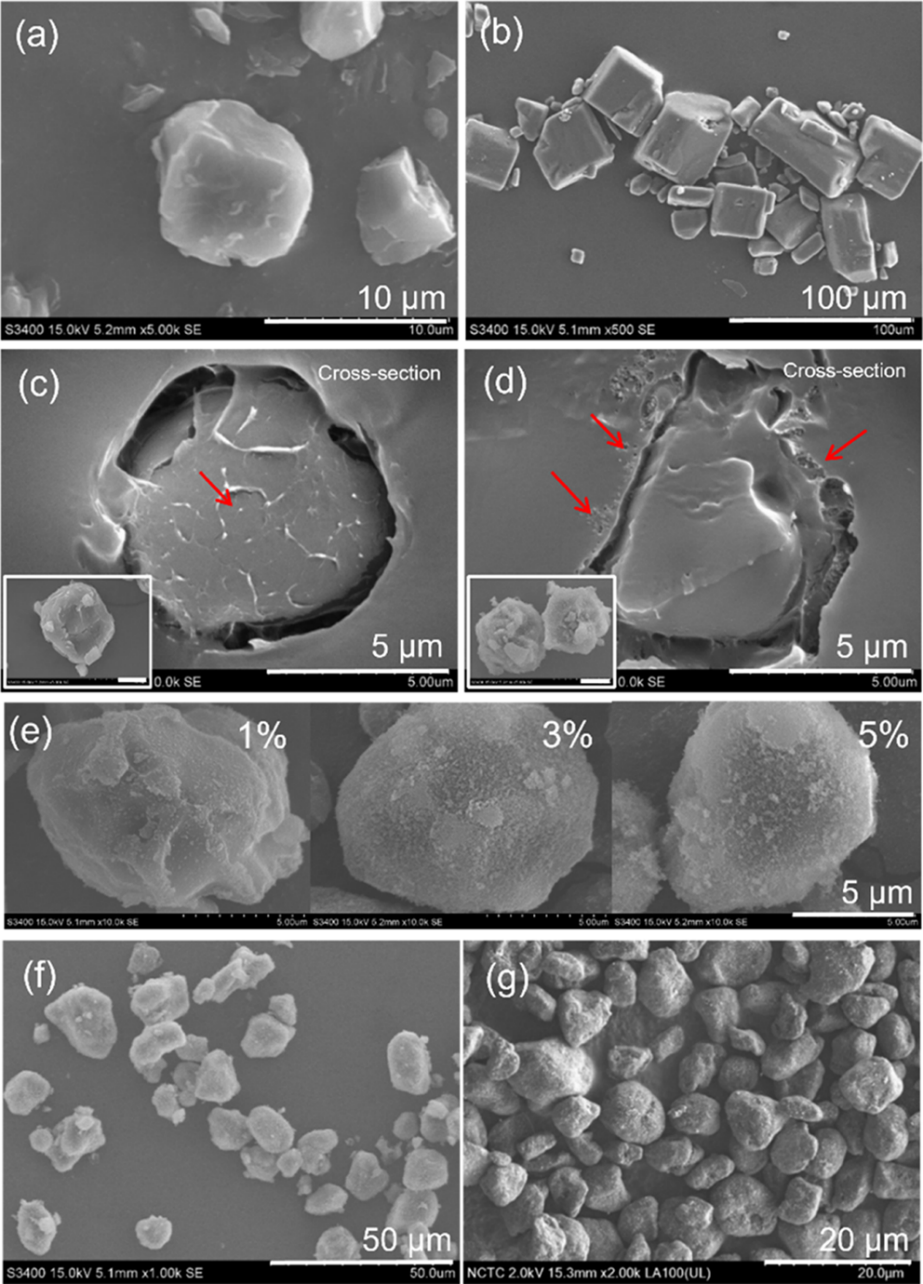
SEM morphologies of (a) R, (b) AA powders, (c) 10C-R (outside and cross section), (d) 10C-R treated with silica (outside and cross section), (e) XR202-10C-R treated with silica 1, 3, 5%, (f) printable encapsulated AA in this study, and (g) commercial toner.

The morphologies of both the outer surface and cross sections of R, AA, 10C-R, and XR202-10C-R were studied using SEM measurements. The results demonstrated that dry-milled rice flour (R) (Figure 3(a)) and 10C-R had similar structures to the initial granules without the presence of free AA crystals (Figure 3(b,c)). Using this process, AA miscible blends in starch and binds well on the surface of granules or generates new particles in the system. We observed a granule of starch within the structure. In addition, loose adhesion of nanosilicas was found on the outer surface of 5R202-10C-R, as indicated by red arrows (Figure 3(d)). Moreover, the effects of different amounts of silica, including 1, 3, and 5% of the total weight, were studied. Results demonstrate that 1% of nanosilica could not fully cover the surface. Conversely, the surface was consistently covered when the silica content was increased to 5%, as shown in Figure 3(e). Hence, 5% silica was applied in this study. The morphology of the printable encapsulated AA and commercial toner is shown in Figure 3(f,g), respectively.

The average sizes of the samples were studied by using the laser diffraction technique. The results demonstrate that dry-milled rice flour (R) has a poor flow due to its high internal adhesion, preventing it from flowing freely during the experiment. Hence, analyzing the particle size of R with this technique in dry mode proved challenging. However, after the encapsulation process, the flow behavior of 10C-R improved significantly, facilitating analysis. The particle sizes of the samples are shown in Table 3. 10C-R exhibits a unimodal distribution and an average mean size of 5.57 (±0.43) μm. After treating their surfaces with various types of nanosilica, the particle sizes of 5HDK-10C-R, 5R812S-10C-R, and 5R202-10C-R were found to be 6.48 (±0.81), 5.25 (±0.19), and 6.54 (±1.05) μm, respectively. Moreover, we observed that the flow behavior of the samples dramatically depended on the electrostatic charges on their surfaces. The charges of the samples were examined by using electrical single-particle aerodynamic relaxation time (E-SPART) measurements. The procedures were conducted following the same protocol as for the commercial toner, and the results are shown in Table 3. R has positively charged surfaces (1.82 (±0.17) μC/g). After encapsulation, 10C-R showed more positive charges on the surfaces (2.98 (±0.25) μC/g). Treatment of the surfaces with nanosilica resulted in negative charges, and the number of charges on the surfaces depended on the type of silica. Our results demonstrated that AEROSIL R202 provided the highest number of negative charges (−12.31 (±0.55) μC/g).

**Table 3:** Particle Size and Charges (*Q*/*M*) of Samples

samples	median size (μm)	mean size (μm)	mode size (μm)	span (μm)	*Q*/*M* (μC/g)
R	n/a	n/a	n/a	n/a	1.82 (±0.17)
10C-R	5.57 (±0.43)	7.16 (±0.36)	3.56 (±0.29)	1.78 (±0.16)	2.98 (±0.25)
5HDK-10C-R	6.48 (±0.81)	7.82 (±0.54)	4.98 (±1.91)	1.53 (±0.18)	–8.78 (±0.07)
5R812S-10C-R	5.25 (±0.19)	6.54 (±0.07)	4.09 (±1.20)	1.65 (±0.05)	–6.90 (±0.27)
5R202-10C-R	6.54 (±1.05)	7.68 (±0.75)	4.99 (±1.91)	1.33 (±0.29)	–12.31 (±0.55)

The flow behavior of the samples was studied. Table 4 shows that the dry-milled rice flour exhibited unfavorable characteristics when evaluating parameters such as the angle of repose (deg), CI (%), and Hausner ratio. Due to the high intrinsic adhesion, 10C-R exhibited a better flow after the encapsulation process. After treatment with nanosilica, the flow behavior dramatically improved.

**Table 4:** Flow Behavior of Samples

samples	angle of repose (deg)	Bulk density (g/cm^3^)	tap density (g/cm^3^)	Carr’s index (%)	Hausner ratio	evaluated based on Carr’s index (%) and Hausner ratio
R	56.24 (±0.67)	0.428	0.697	38.57	1.62	very, very poor
10C-R	57.20 (±0.80)	0.399	0.630	36.69	1.57	very poor
5HDK-10C-R	43.40 (±0.80)	0.478	0.690	30.76	1.44	poor
5R812S-10C-R	41.19 (±0.32)	0.488	0.713	31.46	1.45	poor
5R202-10C-R	47.00 (±0.09)	0.486	0.679	28.49	1.39	poor

The sample composition, especially the presence of intrinsic heavy metals, was examined by using XRF. The preliminary results indicated the absence of heavy metals in all samples, demonstrating that the main constituents were phosphorus (P), sulfur (S), potassium (K), and silica (Si), as shown in Table S4.

The DSC thermograms (Figure S4(a,b)) reveal the *T*_m_ of pure AA to be 194.8 °C. The *T*_m_ of R is 143.3 °C and is attributed to oligosaccharides, resulting from the dry-milling process. After encapsulation, a peak at 138.4 °C was observed, suggesting the presence of a miscible blend of AA/oligosaccharides in R. Furthermore, the silica on the surface of 5HDK-10C-R prevented heat penetration into the sample, shifting the *T*_m_ from 138.4 to 139.6 °C.

The thermo-oxidative decomposition of the initial, pure components was assessed (Figure S4(c,d)). The profile for pure AA involves a three-stage process: (i) the main stage at 222.7 °C with a mass loss of 40%, followed by stage (ii) at 306 °C with 31% of mass loss, and finally (iii) at 700 °C with 15.91% of mass contribution. Jingyan et al. reported that a decomposition process occurred in the first and second stages. In the first stage, decarboxylation and dehydration occurred, while in the second stage, decarboxylation and decarbonylation were the main decomposition processes. In the third stage, only a slow carbonization process occurred.^48^

The pure R involved a three-stage process: (i) at 74 °C with 15% of mass loss associated with humidity release, (ii) at 257.60 °C with a mass contribution of 36%, and (iii) at 625 °C with 18% of mass loss correlated to the decomposition of the previously formed carbonaceous residue.

For 10C-R, the first decomposition stage was found to occur over a range of 50 to 150 °C attributed to the release of bound water molecules. The increasing *T*_d_ of the main stage at 297.70 °C was demonstrated to correlate with the protection against thermal decomposition achieved by employing a wall material (rice starch) in the encapsulation process. The main *T*_d_ state of 5HDK-10C-R was observed at 296.70 °C, suggesting that the silica nanoparticles on their surface could not prevent heat dissipation within the particles. The thermal decomposition temperatures of all samples are listed in Table S5.

### 3.2 Printing Ability of Encapsulated AAs in Rice Flour

**Figure 4:**
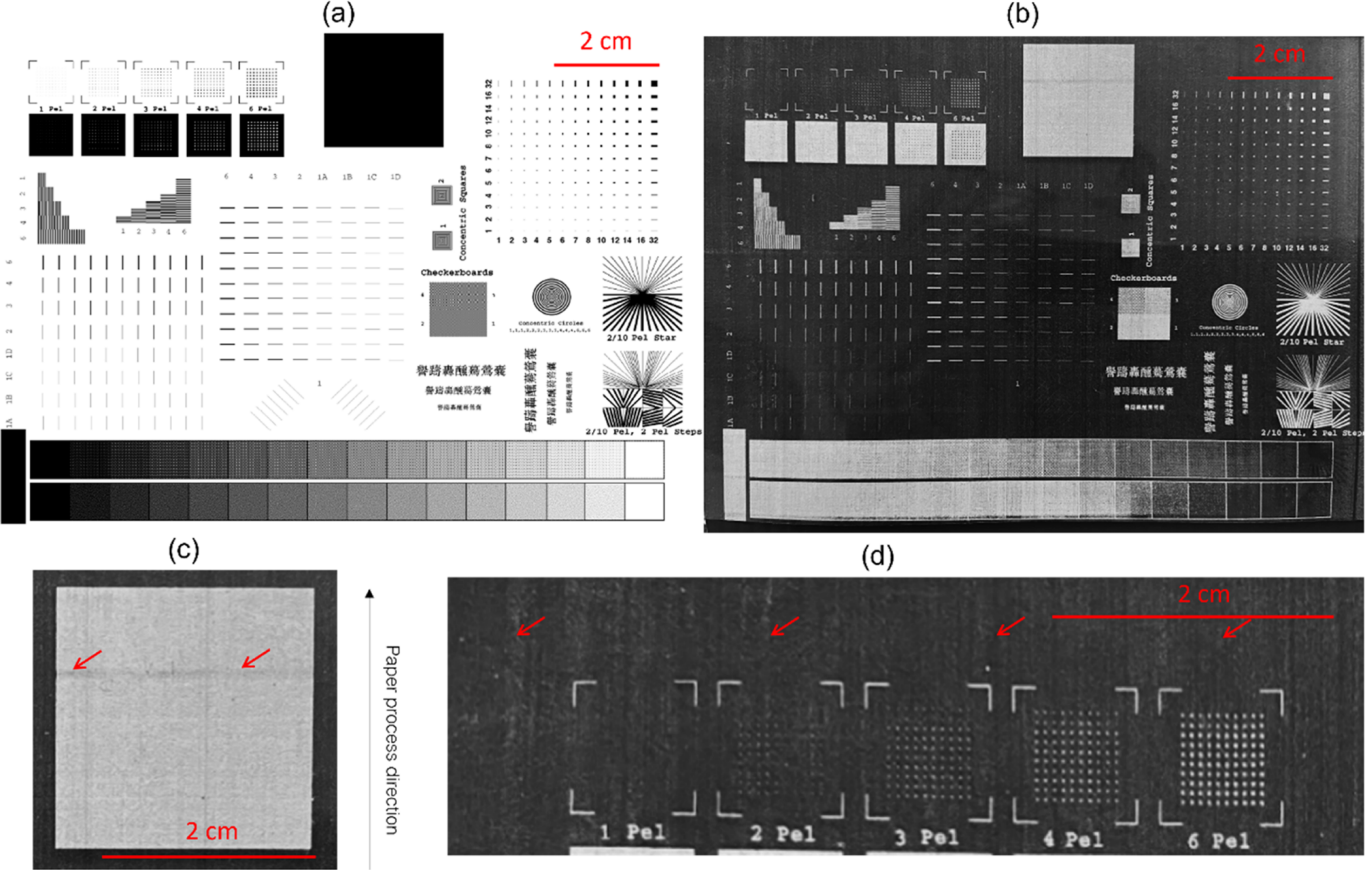
(a) Picture of an original artwork (.pdf), (b) picture of printed artwork, (c) defect in banding, and (d) defect in the background.

In this study, the printing ability of encapsulated AAs in rice flour was assessed using a commercial laser printer (Brother HL-2130). 5R202-10C-R was selected due to its high *Q*/*M* values. Hence, 10 g of 5R202-10C-R was placed into the clean cartridge without further modification. For easy visual inspection of the artwork defects, a clear poly(ethylene terephthalate) (PET) sheet was used as a printing substrate. During the printing process, the laser beam created positive charge patterns on the organic photoconductor (OPC) drum corresponding to the original artwork in Figure 4. Negatively (−) charged powders were deposited on the positively (+) charged areas on the OPC drum and transferred to a PET sheet after the discharging process. Then, they were pressed by a heat roller at 150 °C and 70 psi for a short time (ms) to fix the powders on the PET substrate. The initial artwork (in .pdf format) is shown in Figure 4(a). It comprises dots and lines of different sizes and thicknesses, both along and perpendicular to the printing direction. We found that encapsulated powders created a good pattern, as seen in Figure 4(b). However, an incomplete melt of 5R202-10C-R and some printing defects, such as linear bandings perpendicular to the printing direction (Figure 4(c)) and scattering of powders in the undefined image area, were found (Figure 4(d)). The latter defect was caused by uneven charges on the powder surfaces. No ghosting defect was observed on the printout image. The amount of encapsulated particles per area is 0.28 mg/cm^2^, and the RGB color code is 0,0,0.

### 3.3 % Release of AAs from Encapsulated Samples

In this study, AAs were trapped in solid forms, making it challenging to transfer them directly to the membrane. Therefore, we designed a carrier emulsion system to improve topical delivery. This was done based on the assumption that the carrier emulsion had a suitable polarity to dissolve the AAs from the microcapsules and facilitate their passage through the lipid barrier of the membrane.

A skin permeability test using an artificial membrane (Strat-M) was performed. Initially, a permeability test was conducted using six carrier formulations prepared with three different enhancers, each having a distinct log *P* value. These enhancers included glycerin (G, log *P* = −1.9), pentylene glycol (P5G, log *P* = −0.08), and diethylene glycol monoethyl ether (DEGEE, log *P* = 0.64) at concentrations of 1 and 5% in water. Unfortunately, AAs did not permeate through the Strat-M membranes. This lack of permeability is attributed to the highly hydrophobic nature of the Strat-M membranes, primarily poly(ether sulfone) (PES), permitting only chemical substances with a log *P* between −0.131 and 6.9 to transmit through them. As AAs exhibit a log *P* of −2.15, penetration proves to be challenging for them. Alkilani et al. reported that nonionic surfactants, such as poly(ethylene glycol) sorbitan monolaurate (Tween 20; T20), oleic acid (OA), and diethylene glycol monoethyl ether (DEGEE), improved the permeability of AAs through Strat-M.^40^

Therefore, three trinary phase diagrams were obtained by maintaining a fixed ratio of 1:1 between T20 and OA. In total, 23 formulas of three enhancers were prepared to study the effect of water, the type of enhancers, and the amount of T20/OA on the permeability. Three formulations were selected, including formulations #8, #10, and #17. Formulation #8 consists of enhancer/T20/OA/DI water in a ratio of 20/10/10/60. Formulation #10 is composed of enhancer/T20/OA/DI water in a ratio of 60/10/10/20, while formulation #17 has a composition of enhancer/T20/OA/DI water in a ratio of 20/30/30/20. (Figure S5(a)). All formulations were prepared by using a low-energy method. The appearances of the prepared emulsions are shown in Figure S5(b). To clarify the types of carrier emulsion, an oil phase was stained with Nine Red dye, whereas the water phase was stained with Fit C dye. Then, the emulsions were excited at 512 nm. The phases of the emulsions are shown in Figure S5(c).

We found that different types of enhancers highly affected gel formation and type of emulsion. Figure S5(b) in the Supporting Data shows that glycerin afforded an opaque emulsion in G8, G10, and G17. Moreover, a water-in-oil (O/W) macroemulsion (particle size ∼1–10 μm) occurred in this system. This observation may be attributed to glycerin, which has a log *P* value of −1.9 (log *P* < 0, suggesting an affinity for the water phase). Glycerin likely formed a miscible phase with water, potentially acting as a continuous phase. Conversely, OA acted as an oil phase surrounded by T20 as a nonionic surfactant that can generate macroemulsions. Pentylene glycol (P5G) afforded yellow clear microemulsions in P8, P10, and P17. However, the exact type of emulsion could not be distinguished using a light microscope. This uncertainty arises from the properties of pentylene glycol, which has a log *P* of −0.08 and an HLB value of approximately 8.2,^49^ placing it in a transitional range between water-soluble and oil-soluble characteristics. Hence, pentylene glycol acted as a cosurfactant to stabilize oil droplets. When the amount of P5G increased from 20 to 60, a yellow clear microemulsion was thermodynamically formed. According to Alany et al., the requirements of the cosurfactant molecule to produce a balanced microemulsion were an HLB value of 7.0–8.0, a carbon backbone of 4–6 atoms, and a log *P* value of 0.2–0.9.^50^ DEGEE has a log *P* of 0.64 (log *P* > 0 oil phase) and an HLB of 4.2, miscible with the oil phase. An oil-in-water (O/W) macroemulsion was observed for DEGEE/T20/OA/DI water at a ratio of 20/10/10/60 (T8), while a water-in-oil (W/O) emulsion was detected when using DEGEE/T20/OA/DI water at a ratio of 20/30/30/20 (T17). A yellow, clear microemulsion was observed for DEGEE/T20/OA/DI water at a ratio of 60/10/10/20 (T10).

Then, the pH, conductivity, viscosity, type, stability of the emulsions, and solubility of AAs in carrier emulsions were studied. The results are shown in Table 5. The pH of all carrier formulas was between 4 and 5, which was reported to be suitable for skin application.^51^ The viscosity of the carrier emulsions increased when the content of T20/OA increased owing to the high intrinsic viscosity of T20 (∼250–450 cP). Emulgel formation and agglomeration of oil droplets in a water medium, resulting in a high viscosity of approximately 226.16 cP, were observed when glycerin was used as an enhancer (G17). After centrifuging at 2260*g* for 10 min, the gel became unstable (Figure S5(b)). The conductivity of the carrier emulsion increased with increasing water content, with the exception of G8. The gel formation in this sample retarded the movement of ions, resulting in a low conductivity. Therefore, clarifying the types of carrier emulsions by investigating their conductivity proved challenging.

**Table 5:** Formulations of Carriers, pH, Conductivity, Viscosity, Type of Emulsion, Stability, and Solubility of AA in Carriers

carriers code	enhancer	formulations of carriers enhancer/T20/OA/DI	pH	conductivity (μs/cm)	viscosity (cP) at 25 °C	type of emulsion	stability	solubility of AA (mg/mL)^a^
G8	G	20/10/10/60	4	4.57 (±0.11)	183.66 (±3.74)	o/w	stable	171.89 (±1.47)
G10	G	60/10/10/20	4–5	9.78(±0.27)	69.99 (±1.38)	o/w	stable	136.91 (±5.64)
G17	G	20/30/30/20	4–5	10.08 (±0.07)	226.16 (±3.79)	o/w	unstable	3.27 (±0.79)
P8	P5G	20/10/10/60	4	43.44 (±0.14)	19.99 (±0.06)	n/a	stable	171.08 (±0.46)
P10	P5G	60/10/10/20	4	14.58 (±0.19)	26.13 (±0.01)	n/a	stable	97.97 (±3.82)
P17	P5G	20/30/30/20	4	12.29 (±0.02)	79.25 (±1.78)	n/a	stable	87.26 (±1.44)
T8	DEGEE	20/10/10/60	4	43.44 (±0.14)	15.18 (±0.09)	o/w	stable	21.86 (±15.33)
T10	DEGEE	60/10/10/20	5	11.82 (±0.07)	15.89 (±0.10)	o/w	stable	157.29 (±10.74)
T17	DEGEE	20/30/30/20	4–5	10.54 (±0.03)	96.13 (±0.51)	w/o	stable	96.49 (±1.96)

a Note: After centrifuging at 2260*g* for 10 min, solubility of AAs in pure water = 330 mg/mL and solubility of AAs in pure glycerin = 10 mg/mL.

The solubility of AA powders in nine carrier formulations was investigated by using the DPPH method. A 400 mg (excess) of AA was added to 1 mL of carrier solution at room temperature for 1 h (Figure S6(a)). Notably, T20 did not show scavenging activity on DPPH.^52^ However, OA had a double bond inside its structure, potentially affecting the total antioxidant activity. To reduce the interference of OA, a carrier solution was used as the blank solution. AA in G8 exhibited a high solubility of 171.89 (±1.47) mg/mL, and the AA powder was dissolved well in both pure glycerin (10 mg/mL) and water (330 mg/mL). However, the lowest solubility of the AA powder was found in G17, whereas T20 and OA cannot dissolve AAs. Moreover, owing to its high viscosity of 226.16 cP, the AA behaved like Maple Syrup. Therefore, it was unable to blend effectively with AA powders by using a rotary mixer (Multi Bio RS-24, Biosan) at 50 rpm. Conversely, P8 and T10 demonstrated the ability to dissolve AAs, showing that the solubility of the AAs was highly dependent on the composition and viscosity of the emulsion.

To study the effect of hydrophobic silica on the release behavior of AAs from encapsulation, 10C-R and 5R812S-10C-R were selected. Hydrophobic nanosilica was used to minimize the reduction of negative electrical charges on the surfaces through moisture. However, we found that treating the surface with hydrophobic silica dramatically affected the wettability of the encapsulated AAs. This was clearly observed when we used water as a carrier. In addition, our results demonstrate that silica with a higher carbon content showed a lower %release of AAs. 10C-R, 5HDK-10C-R, 5R812S-10C-R, and 5R202-10C-R provided a %release of 102.3(±4.59), 100.42 (±2.14), 65.75(±4.9), and 52.94 (±12.4)%, respectively. However, pure water could not transmit AAs through Strat-M.

The %release values of encapsulated AAs in 10C-R and 5R812S-10C-R with nine carrier formulations are shown in Figure S6(b). The nine formulations of emulsion carriers showed a lower %release than pure water because of the solubility limit of short-chain starch within the emulsion. The results of 10C-R demonstrated that the type of carrier dramatically affected the %release. G8, G10, and G17 showed a high %release of AAs, although G17 provided a low solubility of AA powder. This may be explained by the fact that glycerin could dissolve short-chain starch and release AAs, which were entrapped inside the particle. The effects of hydrophobic silica on % release were observed in G17 and T17.

### 3.4 Topical Delivery of AAs to Strat-M

Next, the enhanced topical delivery of carrier emulsions was studied. The steady-state flux (*J*_ss_, μg/h/cm^2^), permeability coefficient (*k*_p_, cm/h), and diffusion coefficient (*D*, cm^2^/h) are shown in Table 6. The results showed that the steady-state flux strongly depended on the water content in the formulation. A high water content restricted the penetration of AAs into and across Strat-M. An increase in T20 and OA contents increased the steady-state flux of AA across Strat-M. The most effective enhancer for our system was DEGEE. Furthermore, hydrophobic silica on the particles helped to increase the steady-state flux of AAs to Strat-M. This can be explained by the simple observation that hydrophobic silica functions as an emulsifier^53^ and helps to stabilize water in W/O or oil droplets in O/W. The T17 formulation demonstrated the highest permeability of AAs from 10C-R and 5R812S-10C-R. Moreover, we found that the steady-state flux of AAs did not depend on solubility and %release.

**Table 6:** Permeation Parameters for Penetration of Ascorbic Acid (AA) across Strat-M in the Presence of Penetration Enhancer

samples	enhancer	carrier code	carrier formulations: enhancer/T20/OA/DI	steady-state flux (*J*_ss_, μg/h/cm^2^)	permeability coefficient (*k*_p_, cm/h)	diffusion coefficient (*D*, cm^2^/h)
10C-R	P3G	G8	20/10/10/60	undetected	undetected	undetected
10C-R	P3G	G10	60/10/10/20	7.77 (±0.82)	7.77 × 10^–4^	2.42 × 10^–3^
10C-R	P3G	G17	20/30/30/20	4.62 (±1.28)	4.62 × 10^–4^	1.4 × 10^–3^
5R812S-10C-R	P3G	G17	20/30/30/20	9.75 (±0.44)	9.75 × 10^–4^	3.0 × 10^–3^
10C-R	P5G	P8	20/10/10/60	3.13 (±0.48)	3.13 × 10^–4^	0.98 × 10^–3^
10C-R	P5G	P10	60/10/10/20	5.65 (±2.36)	5.7 × 10^–4^	1.77 × 10^–3^
10C-R	P5G	P17	20/30/30/20	4.18 (±0.10)	4.18 × 10^–4^	1.30 × 10^–3^
5R812S-10C-R	P5G	P17	20/30/30/20	8.40 (±0.64)	8.40 × 10^–4^	2.62 × 10^–3^
10C-R	DEGEE	T8	20/10/10/60	1.55 (±0.21)	1.55 × 10^–4^	0.48 × 10^–3^
10C-R	DEGEE	T10	60/10/10/20	6.59 (±1.34)	6.59 × 10^–4^	2.06 × 10^–3^
10C-R	DEGEE	T17	20/30/30/20	8.44 (±0.47)	8.44 × 10^–4^	2.64 × 10^–3^
5R812S-10C-R	DEGEE	T17	20/30/30/20	10.04 (±0.58)	10.04 × 10^–4^	3.13 × 10^–3^

### 3.5 Cytotoxicity Assay

**Figure 5:**
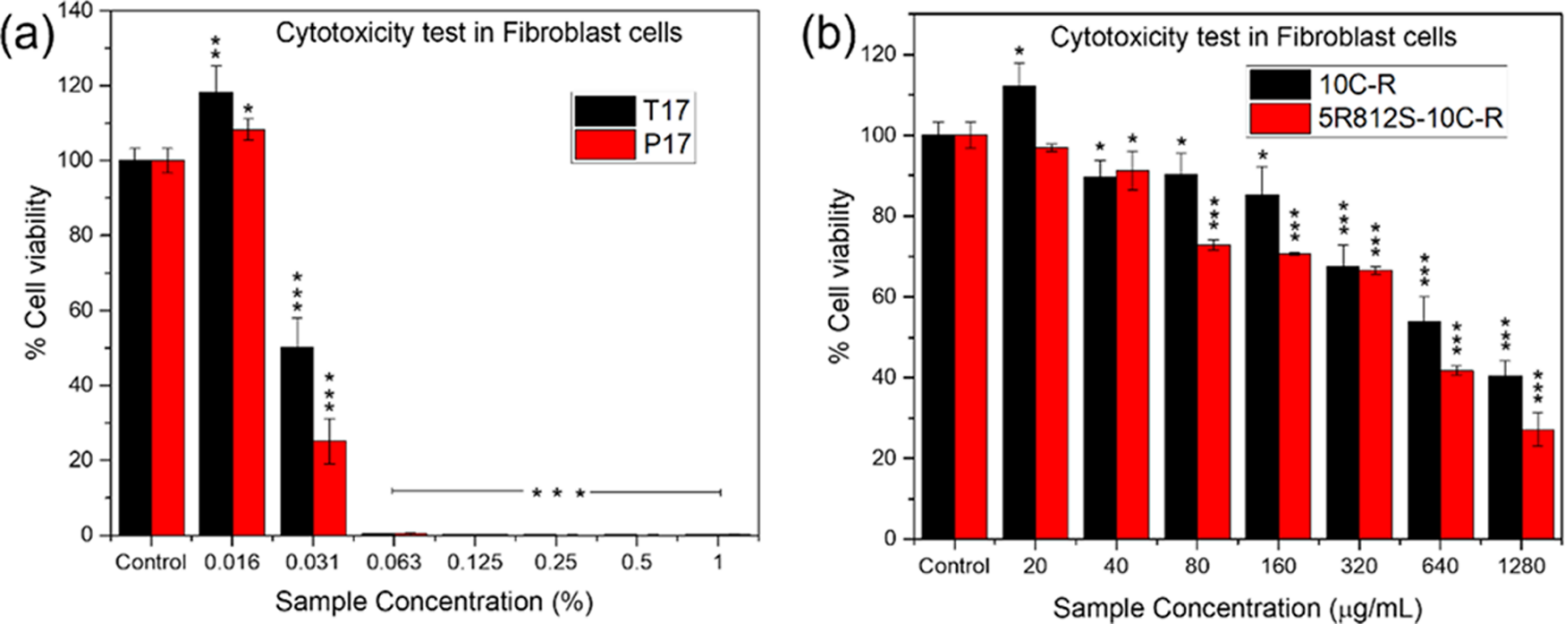
Cytotoxicity in fibroblast cells (a) in carrier solution T17 and P17 and (b) 10C-R and 5R812S-10C-R. Data are expressed as mean ± SD (*n* = 3). *p < 0.05, **p < 0.01, and ***p < 0.001.

The cytotoxicity was studied in fibroblast cells. The 10C-R- and 5R812S-10C-R-encapsulated powders were selected. Due to the instability of G17, two formulations, including T17 and P17, were chosen. The toxicity test results of the samples on fibroblast cells are shown in Figure 5 and Table 7. The 10C-R and 5R812S-10C-R samples exhibited low cytotoxicity because of their high IC_50_ values. The cytotoxicity values were 467.79 μg/mL for 10C-R and 472.78 μg/mL for 5R812S-10C-R. Among the carriers, the T17 (T/T20/OA/DI = 20/30/30/20) and P17 (P/T20/OA/DI = 20/30/30/20) samples had IC_50_ values of 0.0307 and 0.0289%, respectively.

**Table 7:** Inhibitory Concentration 50% (IC_50_) in Fibroblast Cells

samples	IC_50_
10C-R	467.79 μg/mL
5R812S-10C-R	472.78 μg/mL
T17 (T/T20/OA/DI = 20/30/30/20)	0.0307%
P17 (P/T20/OA/DI = 20/30/30/20)	0.0289%

### 3.6 Biocompatibility Assay

**Figure 6:**
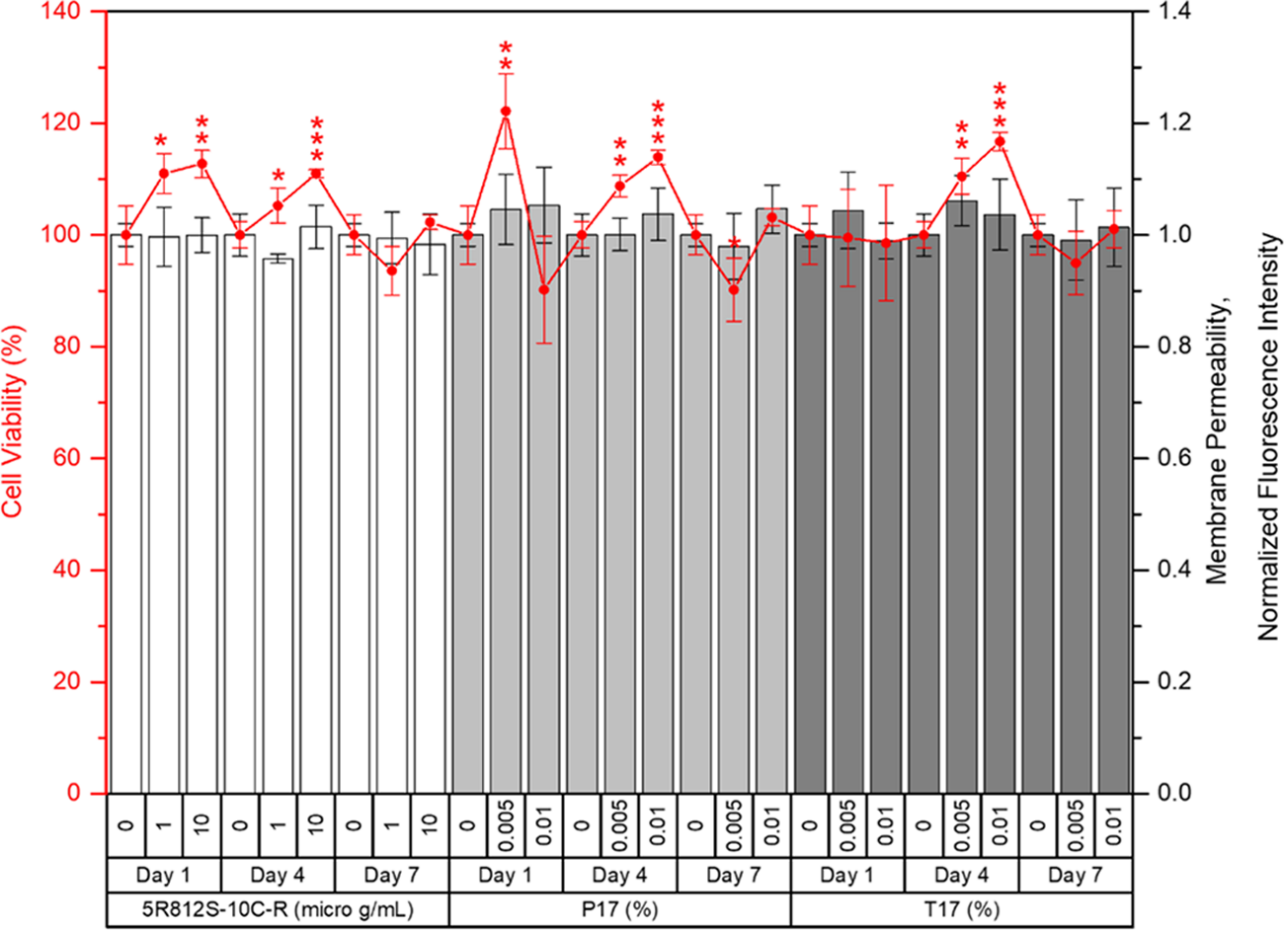
Biocompatibility in fibroblast cells of 5R812S-10C-R, T17, and P17. Data are expressed as mean ± SD (*n* = 4). *p < 0.05, **p < 0.01, and ***p < 0.001.

The encapsulated powder 5R812S-10C-R and 2 carriers, T17 and P17, at the concentrations that gave a percentage of viability more than 80% were selected to study their biocompatibility assay in human fibroblast cells for 7 days without changing the medium. The biocompatibility of treated cells was followed in terms of cell viability by tracking the amount of ATP representing the metabolically active cells and plasma membrane permeability by tracking the impermeant dye to healthy cells. For the encapsulated powder 5R812S-10C-R at 1 and 10 μg/mL, the cell viability was increased significantly on days 1 and 4 compared to the untreated cells, indicating their beneficial effect of cell proliferation. This proliferation effect was slightly decreased on day 7, whereas their membrane permeability was still in normal condition compared to the untreated cells. T17 and P17 carriers at a concentration less than 0.01% could promote and maintain cell viability >90% for 7 days without significant changes in the membrane integrity (Figure 6). This data confirmed the biocompatibility of 5R812S-10C-R, T17, and P17 in human skin cells.

### 3.7 In Vivo Skin Irritation Study

To determine the acute irritation potential of the formulation and carriers, a single patch test was performed on 33 adult subjects. The average ages of tested subjects were 42.91 ± 11.42 years with a male/female ratio of 6/27. A patch containing the samples was applied on the scapular part of the back for 48 h and read 30 min and 24 h after patch removal. The obtained MCII values were graded by a dermatologist based on the occurrence of erythema and edema, as reported in Table 8. The MCII values of the 5R812S-10C-R formulation and T17 and P17 carriers were lower than 0.25, indicating that these tested samples were nonirritating.

**Table 8:** Mean Cumulative Irritation Index (MCII) of the Formulation and Carriers

samples	MCII
5R812S-10C-R	0.18
T17 (T/T20/OA/DI = 20/30/30/20)	0.08
P17 (P/T20/OA/DI = 20/30/30/20)	0.06

## 4 Conclusions

We have successfully developed a method for preparing printable microencapsulated AAs for personalized topical delivery using laser printing technology. Specifically, we present the characteristics of dry-milled rice flour, including its morphology and composition, and describe an appropriate encapsulation method that can preserve the printable structure and stability of AA. We then treated the surface of the encapsulated powders with hydrophobic silica and obtained an average particle size of approximately 6–7 μm with negative charges on the surfaces. The flowability of the encapsulation products was improved, and the encapsulated powders were successfully fabricated by using a commercial laser printer. We observed several printing defects, including linear bands and the scattering of powders in the background. However, the steady-state flux obtained from transdermal testing revealed two effective formulations, P17 and T17, with pentylene glycol (P5G) and diethylene glycol monoethyl ether (DEGEE) as enhancers, respectively. The steady-state flux of P17 and T17 was equal to 8.40 (±0.64) and 10.04 (±0.58) μg/h/cm^2^, respectively. We also studied the cytotoxicity of the encapsulated product and carrier in fibroblasts and observed low cytotoxicity. Furthermore, nonirritation results after a single patch test on 33 adult subjects are reported. We believe that the printable-microencapsulated active ingredients for personalized topical delivery presented in this work facilitate the creation of precision skin therapies and improved cosmeceutical methods.

## Supporting Information Available

Details regarding specifications of hydrophobic silica; flow characteristics, properties of materials (including log *P*, hydrophile–lipophile balance (HLB), and viscosity); photographs (including encapsulated samples and carrier emulsions); FTIR spectra of rice flour both of the soluble and insoluble parts; results of the DSC and TGA thermograms of the starting materials and encapsulated samples, XRF, and results of the solubility of the AAs and encapsulated AAs in carriers (PDF)
