# Supplementary material for: Printable-Microencapsulated Ascorbic Acid for Personalized Topical Delivery
Source: ACS Appl Bio Mater. 2023 Nov 20;6(12):5385–98. doi: 10.1021/acsabm.3c00648 (PMC10731657; doi:10.1021/acsabm.3c00648)
Supplement: Supplementary file 1 — mt3c00648_si_001.pdf [file mt3c00648_si_001.pdf]

Supporting Information

Printable-microencapsulated ascorbic acid for personalized topical delivery

Lapporn Vayachuta[a]*, Meyphong Leang[a], Jareerat Ruamcharoen[b], Raweewan Thiramanas[a], Sagaw Prateepchinda[a], Panida Prompinit[a], Sakkarin Du-a-man[a], Sutthinee Wisutthathum[c], Neti Waranuch[c]

[a] National Nanotechnology Center (NANOTEC), National Science and Technology Development Agency (NSTDA), Khlong Luang, Pathum Thani 12120, Thailand
[b] Faculty of Science and Technology, Prince of Songkla University, Muang, Pattani 94000, Thailand
[c] Cosmetics and Natural Products Research Center, Faculty of Pharmaceutical Sciences, Naresuan University, Phitsanulok 65000, Thailand
*Corresponding to Lapporn Vayachuta, E-mail address: lapporn@nanotec.or.th

Table S1 Specifications of hydrophobic silica

Grades	BET surface area [m^2/g]	Loss on drying [%]	pH value	Carbon Content [%]
AEROSIL® R202	80-120	≤ 0.5	4.0-6.0	3.5-5.0
AEROSIL® R812S	220 ± 25	≤ 0.5	5.5-9.0	3.0-4.0
HDK-H15	130-170	≤ 0.6	3.8-4.8	0.8

Table S2 Flowability characteristics based on Angle of Repose, Hausner Ratio, and Carr's Index (CI)

Angle of Repose	Hausner Ratio	CI (%)	Flow character
25-30	1.00-1.11	≤10	Excellent
31-35	1.12-1.18	11-15	Good
36-40	1.19-1.25	16-20	Fair
41-45	1.26-1.34	21-25	Passable
46-55	1.35-1.45	26-31	Poor
56-65	1.46-1.59	32-37	Very poor
>66	›1.60	>38	Very. very poor

Table S3 Abbreviations, chemical formula, LogP, Hydrophile-lipophile balance (HLB) and viscosity of materials

Active/enhancer	Abbreviations	Chemical formula	LogP[37]	HLB	Viscosity (cP)
Ascorbic acid	AA	$C_6H_8O_6$	-1.58	-	-
Water	DI	H_2O	-0.5	-	0.890
Oleic Acid	OA	$C_{18}H_{34}O_2$	7.68	1	27.64
Tween 20	T20	$C_{26}H_{50}O_{10}$	2.75	16.7	250-450
Glycerin	G	$C_3H_8O_3$	-1.9	-	934
Pentylene glycol	P5G	$C_5H_{12}O_2$	-0.08	8.43	-
Diethylene glycol monoethyl ether	DEGEE	$C_6H_{14}O_3$	0.64	4.2	1.7

Table S4 Composition of samples

Samples	Si	P	S	K
R	-	15.06 (±0.25)	42.58 (±0.18)	42.36 (±0.40)
10C-R		15.09 (±0.26)	42.27 (±0.55)	42.64 (±0.76)
5HDK-10C-R	72.78 (±0.17)	6.57 (±0.18)	13.47 (±0.12)	7.18 (±0.24)
5R812S-10C-R	72.86 (±0.21)	6.39 (±0.20)	13.87 (±0.32)	6.88 (±0.19)
5R202-10C-R	71.78 (±0.55)	6.53 (±0.28)	13.97 (±0.19)	7.72 (±0.22)

Table S5 TGA under O_2

Samples	T_{Onset} (°C)	T_{Peak} (°C)	Weight loss at 75-100 °C	Residual at 700 °C (%)
AA	190.9	222.70	-	15.91
R	257.60	305.10	-	-6.29
10C-R	247.50	297.70	-	14.42
5HDK-10C-R	245.50	296.70	-	4.89
5R812S-10C-R	238.20	297.70	-9.19	-2.82
5R202-10C-R	246.00	296.40	-8.09	-10.75
3R202-10C-R	246.30	297.00	-7.96	-3.68
1R202-10C-R	245.40	297.20	-8.30	-15.15

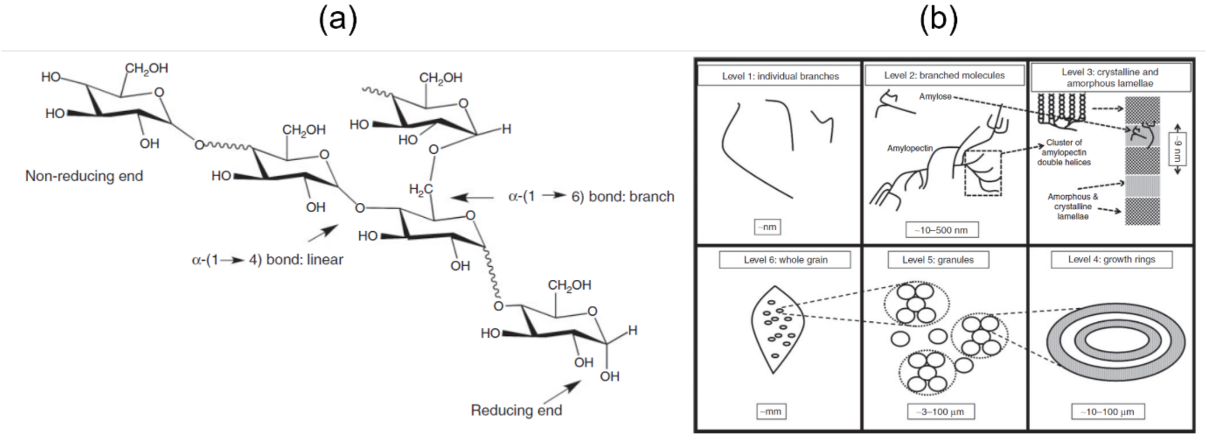

Figure. S1 (a) Chemical structure of amylose and amylopectin in rice flour. (b) The six structural levels of starch, from that of the individual chains to the whole grain.[42]

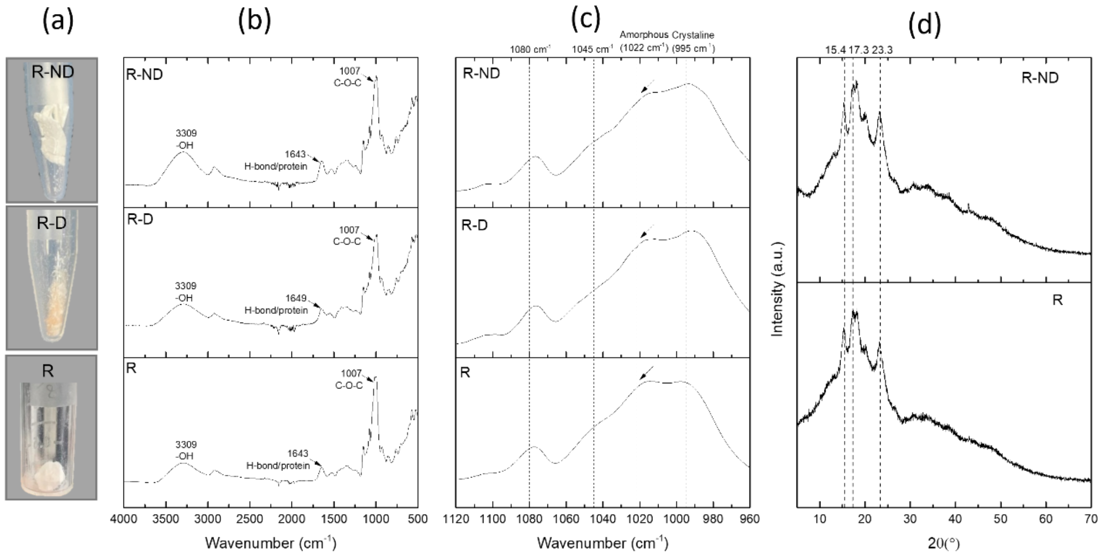

Figure. S2 (a) Photographs of bare rice granules (R), soluble part (R-D), and non-soluble part (R-ND), (b) FT-IR at 500-4000 cm^{-1} and (c) FT-IR at 960-1120 cm^{-1} and (d) XRD spectra of R and R-ND.

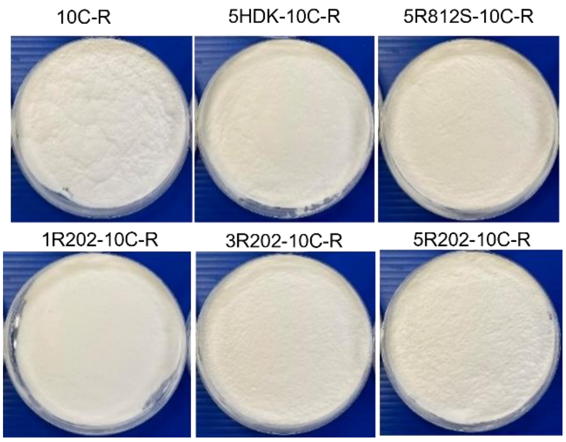

Figure. S3 Photographs of encapsulating particles

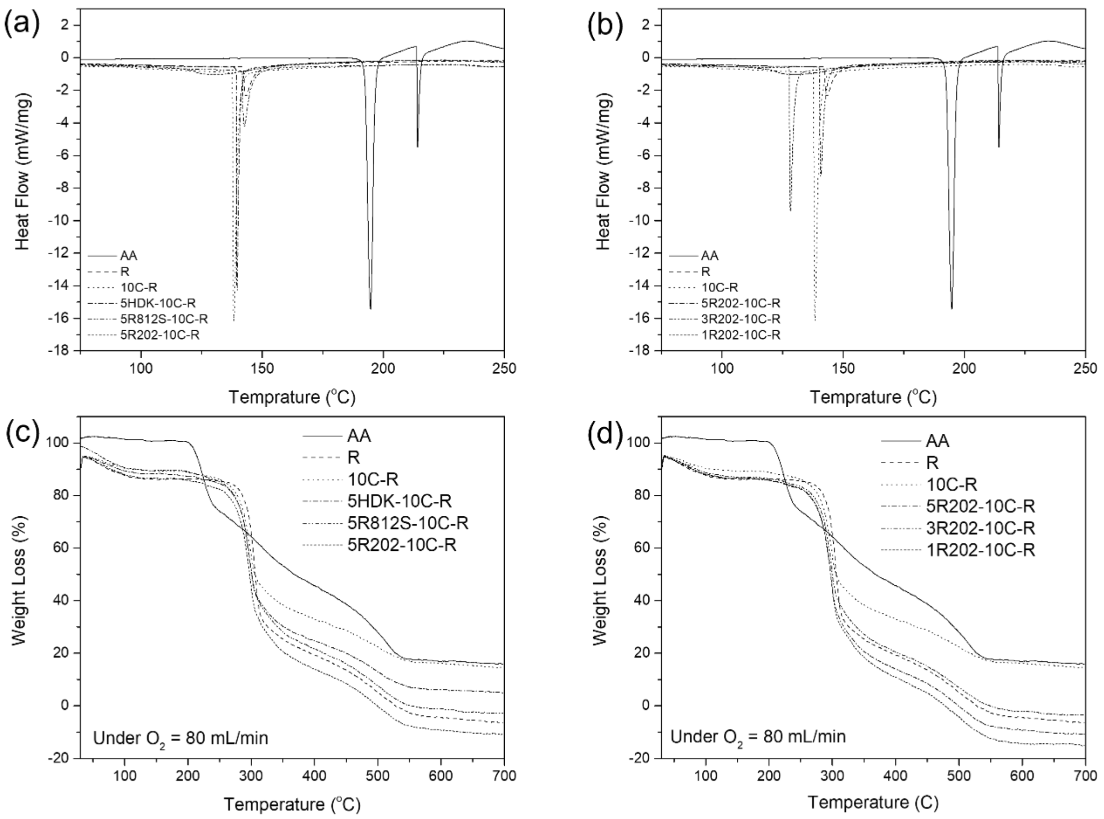

Figure. S4 (a) DSC thermograms of AA, R, 10C-R and 5HDK-,5R812S-,5R202-10C-R (b) DSC thermograms of AA, R, 10C-R and 1R202-,3R202-,5R202-10C-R (c) TGA thermograms of AA, R, 10C-R and 5HDK-10C-R and (d) TGA thermograms of AA, R, 10C-R and 1R202-,3R202-,5R202-10C-R.

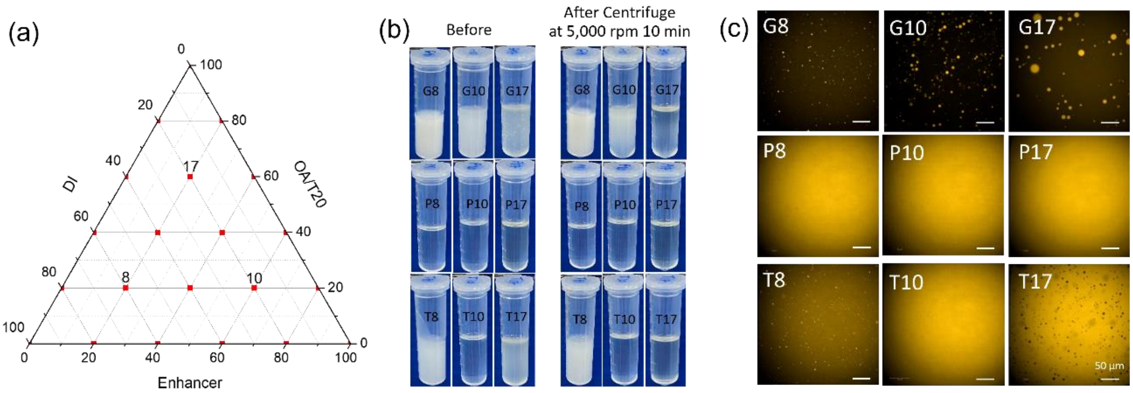

Figure. S5 (a) Trinary phase diagram, (b) appearances of carrier emulsion before and after centrifuge at 5,000 rpm for 10 minutes, and (c) photographs of carrier emulsions after exciting them under 512 nm.

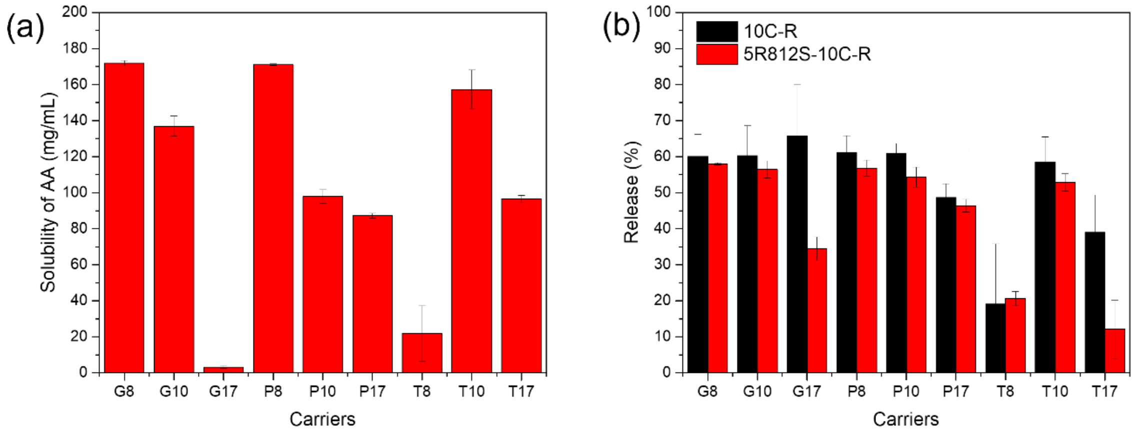

Figure. S6 (a) Solubility of AAs in carriers and (b) release (%) of encapsulated AAs in carriers.
